# Platypnea Orthodeoxia Syndrome of Intra-cardiac Etiology: A Case Report

**DOI:** 10.7759/cureus.92401

**Published:** 2025-09-15

**Authors:** Chelsea Grant, Matthew Lippmann, Azhar A Supariwala

**Affiliations:** 1 Internal Medicine, South Shore University Hospital, Northwell Health, Bay Shore, USA; 2 Cardiology, South Shore University Hospital, Northwell Health, Bay Shore, USA; 3 Cardiology, Northwell Health, Bay Shore, USA

**Keywords:** bubble study, cardiac shunt, echocardiogram, patent foramen ovale, postural hypoxemia

## Abstract

While the prevalence of patent foramen ovale (PFO) is well-established in the literature, the prevalence of platypnea orthodeoxia syndrome (POS), a rare cause of positional hypoxemia, requires further investigation.

An 88-year-old woman presented with respiratory distress and was ultimately diagnosed with POS following multiple cardiac procedures that failed to identify a structural cause of her dyspnea. Outpatient evaluation of oxygen saturations in both sitting and supine positions in addition to supine echocardiography revealed a position-dependent PFO. The patient’s hypoxemia then resolved with shunt closure.

Echocardiography with micro-bubble studies as well as attention to filling pressures should therefore be performed with variation in the vertical axis to rule in POS in cases of dyspnea and hypoxemia of undetermined etiology.

PFO can cause intra-cardiac shunts that appear with changes in position and cause dyspnea via POS. It is ultimately important, however, to understand that not every PFO needs to be closed and that patients should be carefully selected for shunt closure when indicated by right heart hemodynamics.

## Introduction

First described in 1949 by Burchell and colleagues in a patient with post-traumatic arteriovenous malformation (AVM) shunt, platypnea-orthodeoxia syndrome (POS) is a rare cause of postural-dependent hypoxemia defined as a decrease in arterial oxygen pressure by more than 4 mm Hg or a decrease in oxygen saturation >5% during a change from supine positioning to upright positioning [1,2]. Given that hypoxemia involving both cardiac and pulmonic shunts does not resolve with oxygen, the diagnostic approach for POS involves transthoracic echocardiography (TTE) with "bubble study" to evaluate for possible shunt. The appearance of micro-bubbles in the left atrium (LA) within 3-6 cardiac beats after opacification of the right atrium is considered positive for the presence of an intra-cardiac shunt such as a patent foramen ovale (PFO), whereas the appearance of micro-bubbles in the LA after 3-6 cardiac beats indicates intrapulmonary shunting [3,4]. Physiologic right-to-left shunting, however, occurs without hemodynamic or phenotypic deficits during the return of blood products from bronchial arteries to pulmonic veins and during the return of blood flow from thebesian veins to the left ventricle [5]. Elucidation of pulmonary etiologies if TTE and transesophageal echocardiography (TEE) are unrevealing is the next step and may be pursued through pulmonary angiography, contrast-enhanced computed tomography (CT), and ventilation/perfusion (V/Q) scan with technetium-99 labeled macro-aggregated albumin [5]. The definitive treatment of POS is dependent on the underlying etiology, with percutaneous closure of the causative shunt followed by dual antiplatelet therapy for 1-6 months being the reference standard [3]. 

The following case study highlights an intra-cardiac shunt etiology of the uncommon POS.

## Case presentation

**Figure 1:**
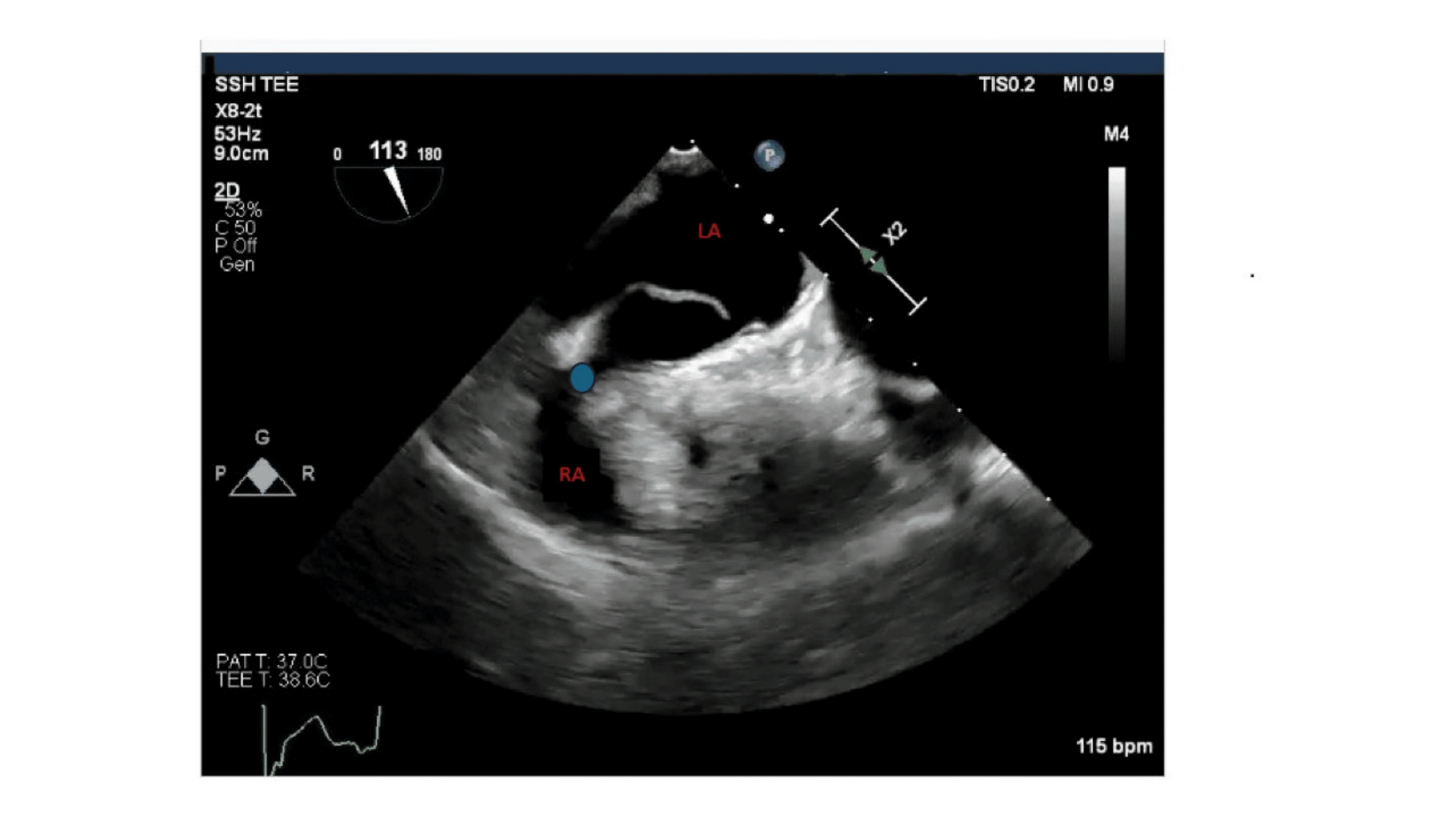
TEE bicaval view depicting PFO (blue circle). The blue circle draws focus to the conduit between the left and right atria or the patent foramen ovale. The photo was captured during a trans-esophageal echocardiogram where a scope was inserted into the patient's mouth and down the esophagus to acquire views of the heart's chambers. The bicaval view of this echocardiogram allows visualization of the left atrium, right atrium, inter-atrial septum, superior vena cava, and inferior vena cava. TEE: Transesophageal Echocardiogram; PFO: Patent Foramen Ovale; RA: Right Atrium; LA: Left Atrium

**Figure 2:**
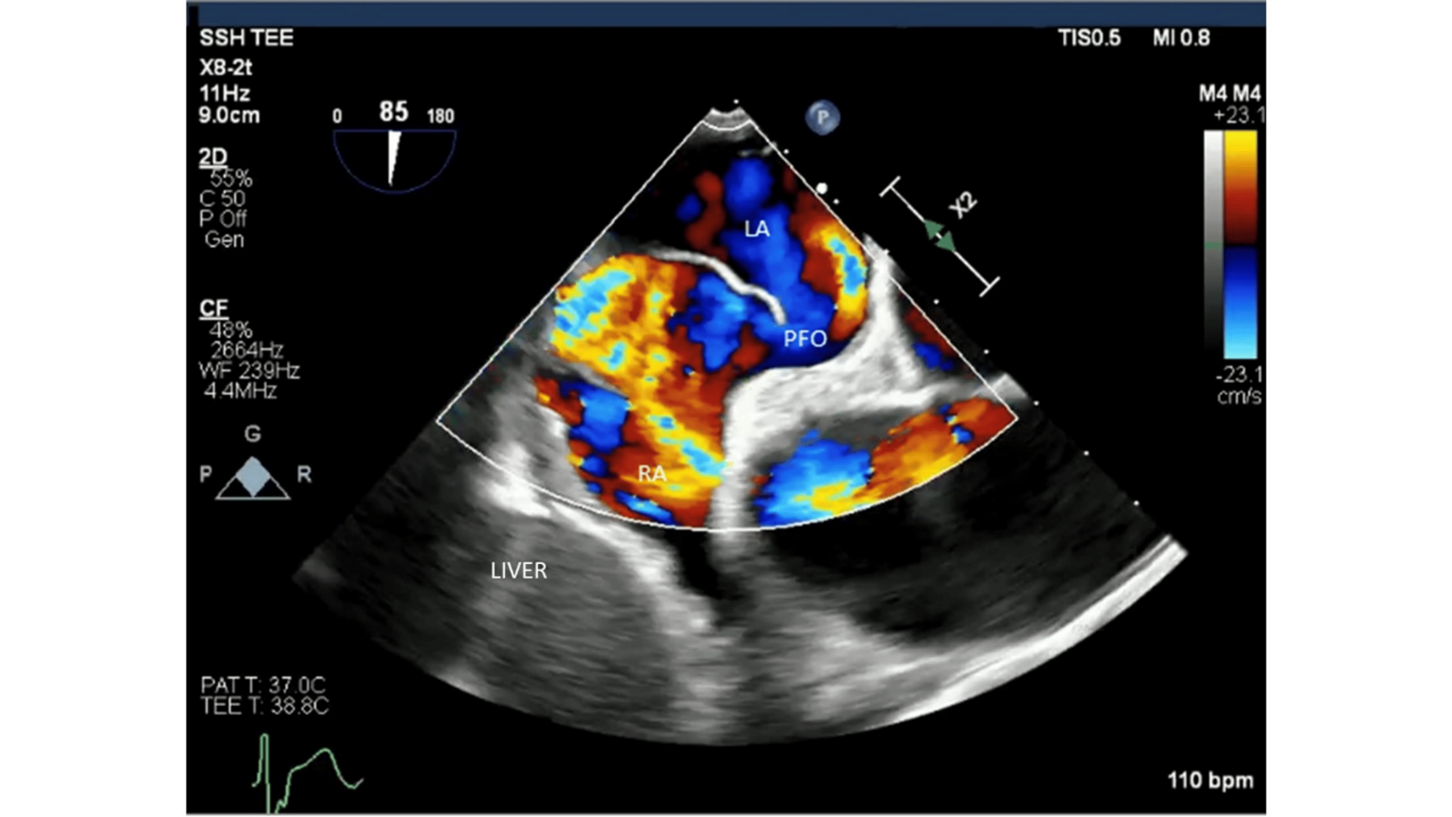
TEE bicaval view with color Doppler depicting PFO. The photo was captured during a trans-esophageal echocardiogram where a scope was inserted into the patient's mouth and down the esophagus to acquire views of the heart's chambers. The bicaval view of this echocardiogram allows visualization of the left atrium, right atrium, inter-atrial septum, superior vena cava, and inferior vena cava. The area within the right atrium with colors yellow, red, and blue indicates an area of high turbulence as blood moves between the high-pressure left atrium to the low-pressure right atrium via the PFO conduit in the color Doppler mode of echocardiogram capture. TEE: Transesophageal Echocardiogram; LA: Left Atrium; PFO: Patent Foramen Ovale; RA: Right Atrium

An 88-year-old woman was brought into the hospital by ambulance for hypoxia. Past medical history is significant for non-obstructive coronary artery disease (CAD), hypertension, type 2 diabetes, and hyperlipidemia. Family history is negative for cerebrovascular accidents (CVAs) and CAD. The patient has no known allergies, and social history is negative for tobacco use and occupational exposure to carcinogens. The patient had been experiencing shortness of breath with exertion for the past 3-4 years prior to presentation. On arrival, her O2 saturations were 80% on room air, which improved to 97% after intervention with 10 liters of oxygen by non-rebreather (reference ranges in Table 1). The heart rate in triage was 50 beats per minute (bpm) with a respiratory rate of 22 breaths per minute. Brain natriuretic peptide and Troponin were negative as were chest radiogram (CXR) and CT angiography. TTE during initial hospitalization demonstrated LVEF 70-75% (reference ranges in Table 2), pericardial fat pad, no pericardial effusion, mild aortic regurgitation, and mild pulmonic regurgitation. Right heart catheterization (RHC) revealed pulmonary artery pressure of 40/17 mm Hg, pulmonary artery wedge pressure (PAWP) of 16 mm Hg, right ventricular (RV) pressure of 43 mm Hg, end-diastolic pressure (EDP) of 12 mm Hg, and right atrial (RA) pressure of 13 mm Hg (reference ranges in Table 3). Two months post discharge, a bilateral venous duplex ultrasound was negative for deep vein thrombosis. After five months post hospitalization, the patient remained on oxygen. During an office visit, saturations were checked with the patient standing, sitting, and supine. A preliminary diagnosis of platypnea-orthodeoxia was made, and a TTE was ordered with “bubble study”. TTE revealed a significant right-to-left shunt when the patient was supine. The shunt was later confirmed with a trans-esophageal echocardiogram (Video 1, Figures 1, 2). The patient underwent atrial level shunt closure with a 30 mm PFO Talisman device six months following initial presentation and was thereafter prescribed aspirin 81 mg and clopidogrel 75 mg for three months. The patient’s symptoms have since improved, and she is currently off supplemental oxygen. 

**Video 1:** TEE Bicaval view with bubble study. Translocation of micro-bubbles from the right to left atria within three cardiac cycles indicates the presence of an intra-cardiac shunt. The video was captured during a trans-esophageal echocardiogram where a scope was inserted into the patient's mouth and down the esophagus to acquire views of the heart's chambers. The bicaval view of this echocardiogram allows visualization of the left atrium, right atrium, inter-atrial septum, superior vena cava, and inferior vena cava. TEE: Transesophageal echocardiogram

**Table 1:** Vital sign reference ranges in a healthy adult mmHg: Millimeters of Mercury units of Pressure; Bpm: Beats per Minute

Vital Sign Reference Ranges in a Healthy Adult > 18 years			
Oxygen (O_2_) Saturation (%)	Arterial Oxygen Pressure (mmHg)	Heart Rate (bpm)	Respiratory Rate (per minute)
92-100	75-100	60-100	12 -20

**Table 2:** Left ventricular ejection fraction reference ranges LVEF: Left Ventricular Ejection Fraction, the fraction of blood ejected by the left ventricle during each cardiac systolic cycle.

Left Ventricle Function	LVEF Reference Ranges (%)
Hyper-dynamic	> 75%
Normal	55-75%
Mildly Reduced	45-54%
Reduced	< 45%

**Table 3:** Right heart catheterization reference ranges RHC: Right Heart Catheterization; mmHg: Millimeters of Mercury Units of Pressure

Right Heart Catheterization (RHC) Reference Ranges in a Healthy Adult > 18 years		
RHC Parameter	Case Patient RHC Results (mmHg)	Reference Ranges (mmHg)
Pulmonary Artery Pressure	40/17	15-30/ 4-12
Pulmonary Artery Wedge Pressure	16	4-12
Right Ventricular Pressure	43	15-30
End Diastolic Pressure	12	0-8
Right Atrial Pressure	13	0-7

## Discussion

The hypoxemia in the setting of POS is either due to (1) intra-cardiac shunt via a PFO, ASD, or a fenestrated atrial septal aneurysm, (2) intra-pulmonary shunt via an AVM, or (3) V/Q mismatch via parenchymal lung disease or hepato-pulmonary syndrome. Intra-cardiac shunts account for approximately 80-87% of POS, with a concomitant anatomical abnormality such as a prominent Eustachian valve, ascending aorta dilatation, thoracic spine kyphoscoliosis, or hemi-diaphragmatic paralysis, predisposing these individuals to right-to-left shunting and postural hypoxia [6,7]. Approximately 30% of adults have a PFO without right-to-left atrial shunt, only shifting to right-to-left shunting with elevated RA pressures secondary to pulmonary thromboembolism, RV myocardial infarction, pneumonectomy, chronic pulmonary hypertension (PH), and tricuspid regurgitation [8]. Distinguishing between the types of shunts is important for diagnostic and therapeutic purposes. The etiology of our patient’s POS was intra-cardiac shunting via PFO with right-to-left shunting. As the patient of this case study hadn't succumbed to a PFO-related stroke and had other causes of hypoxemia definitively ruled out, the Society for Cardiovascular Angiography and Interventions (SCAI) recommends PFO closure for treatment. Although there were reports of adverse events, including the onset of Afib following PFO closure, the majority (76%) of patients reported marked improvement in quality of life following intervention and therefore form the basis for this treatment guideline [9].

Similar to how longstanding left-to-right shunting associated with congenital heart malformations can ultimately lead to pulmonary hypertension and shunt reversal in Eisenmenger syndrome, long-standing pulmonary hypertension in the presence of PFO can lead to right atrial pressures greater than left, leading to a change of shunt direction. PFO is present in approximately 1 in 4 individuals, occurring in 20-34% of the population, and is generally considered benign if presenting with no symptoms [10]. However, in patients with long-standing PH and right heart dysfunction, the shunt direction and flow of blood is from right-to-left, which can cause profound hypoxemia. In both aforementioned scenarios, closure of the shunt is discouraged and can lead to catastrophic consequences with fulminant RV failure [11]. RHC data are vital to rule out both PH and right heart dysfunction before attempting shunt closure. Shunt closure in these scenarios may improve oxygenation but may lead to severe right heart failure. In this patient's case, an RHC showed near-normal pulmonary pressures with normal RV function on echo. For our patient, orthodeoxia was related to anatomically relevant positional deformation pressure changes within the heart, similar to those detected via coronal CT imaging in another case study in Canada (Figure 3) [12].

Pulmonary AVMs would show similar TTE findings with a positive micro-bubble study. However, micro-bubbles are typically seen in the LA after 3 beats (ideally seen coming from pulmonary veins), whereas in an intra-cardiac shunt, micro-bubbles are visualized in the LA in less than 3 beats (ideally seen crossing the inter-atrial septum). A CT scan with IV contrast with PE study protocol is adequate for AVM diagnosis. In most cases, AVMs are focal and may not cause significant hypoxemia. Embolization of these AVMs is usually only needed in cases of transient ischemic attacks/ cerebral vascular accidents (TIA/CVA). However, if large areas of AVMs are present and cause significant hypoxemia, treatment with embolization is indicated. Diffuse pulmonary arterial dilation, as seen with hepato-pulmonary syndrome, on the other hand, will not be seen on a chest CT scan. These AVMs are small, microscopic junctions at the capillary level and are not recognized by a CT scan. A strong clinical suspicion is therefore of paramount importance to diagnose this condition. First, an intra-cardiac shunt is ruled out with an echo showing a positive bubble study after 3 beats. Given that patients with this syndrome usually have porto-pulmonary hypertension, hypoxemia gets worse with treatment for pulmonary hypertension, as the pulmonary vasculature is already vaso-dilated. Treatment of POS caused by hepato-pulmonary syndrome is usually liver transplantation, as hypoxemia is suggestive of poor prognosis in patients with cirrhosis. 

Generally, iatrogenic ASDs that are created during left atrial access with an atrial septostomy heal within 6-8 weeks. The majority of these procedural defects that are created during left atrial pulmonary vein isolation in atrial fibrillation ablation, watchman procedures, and mitral clip procedures do not result in defects greater than 3.5 mm [13]. However, depending upon the tissue character, tissue friability, and procedural techniques, the defects may become larger than expected. In the setting of normal right heart pressures, average caliber defects created will lead to a left-to-right shunt. Depending on the size of the shunt, it can be left to heal on its own, and most often does not require intervention. However, there are situations where acute hypoxemia can occur immediately after septostomy due to a significant right-to-left shunt in patients with more than moderate pulmonary hypertension and where RA pressures are higher than LA pressures. The direction and magnitude of flow across the defect depend on left and right heart pressures as well as the size of the defect. In these cases, keeping the patient hypoxemic until the tissue heals is not an acceptable option, and the defect should be closed immediately to prevent dependence on high-flow oxygen. Because closure of this iatrogenic ASD does not cause acute compensatory pressure changes in the right heart, there is no hemodynamic compromise, and the right heart/pulmonary pressures will continue at the same severity as pre-iatrogenic ASD shunt creation. This is in direct contradiction to patients who have chronic pulmonary hypertension with right heart failure in the setting of a PFO with right-to-left shunt. As discussed above, treatment of PH is the goal in this scenario since closure of the PFO that has been venting the right heart and right atrium in chronic cases can cause a sudden increase in right heart load, causing fulminant RV failure. Nevertheless, before performing atrial septostomy in cases where the patient has severe PH and RV failure, an RHC can confirm the severity of pulmonary pressures. During RHC, the degree of right-to-left shunt can be predicted, and the team of interventional cardiologists should be prepared to close the iatrogenic ASD if the defect size is large and if right-to-left shunt leads to significant hypoxemia. 

## Conclusions

In conclusion, this patient had a PFO that did not cause any symptomatic ramifications until she started having positional deformation of the inter-atrial septum, causing a significant right-to-left shunt with normal pulmonary pressures. Because her PFO had no coexistent pulmonary hypertension by the time of detection, closure of the PFO resolved her platypneic symptoms. This case is unique in that while a longstanding PFO managed to cause right-to-left shunt with supine positioning, it had not been preceded by compensatory increases in pulmonary systolic and right heart pressures that would have precluded PFO vent closure. It is therefore important to understand that not every PFO needs to be closed, and physicians should keep in mind the above-mentioned scenarios to ensure that patients are carefully selected for shunt closure when indicated by right heart hemodynamics. 
